# HACEK Infective Endocarditis: Characteristics and Outcomes from a Large, Multi-National Cohort

**DOI:** 10.1371/journal.pone.0063181

**Published:** 2013-05-17

**Authors:** Stephen T. Chambers, David Murdoch, Arthur Morris, David Holland, Paul Pappas, Manel Almela, Nuria Fernández-Hidalgo, Benito Almirante, Emilio Bouza, Davide Forno, Ana del Rio, Margaret M. Hannan, John Harkness, Zeina A. Kanafani, Tahaniyat Lalani, Selwyn Lang, Nigel Raymond, Kerry Read, Tatiana Vinogradova, Christopher W. Woods, Dannah Wray, G. Ralph Corey, Vivian H. Chu, International Collaboration on Endocarditis Prospective Cohort Study (ICE-PCS) Investigators

**Affiliations:** 1 Department of Pathology, University of Otago, Christchurch and Christchurch Hospital, Christchurch, New Zealand; 2 Microbiology Laboratory, Auckland City Hospital, Auckland, New Zealand; 3 Microbiology Laboratory, Middlemore Hospital, Auckland, New Zealand; 4 Duke Clinical Research Institute, Duke University Medical Centre, Durham, North Carolina, United States of America; 5 Department of Microbiology, Hospital Clinic – IDIBAPS, University of Barcelona, Barcelona, Spain; 6 Infectious Diseases Department, Hospital Universitari Vall d'Hebron, Barcelona, Spain; 7 Clinical Microbiology and Infectious Diseases Service, Hospital General Universitario Gregorio Marañón, Madrid, Spain; 8 Cardiology Department, Maria Vittoria Hospital, Torino, Italy; 9 Department of Clinical Medicine, Infectious and Tropical Diseases, Hospital Clinic – IDIBAPS, University of Barcelona, Barcelona, Spain; 10 Department of Medical Microbiology, Mater Hospitals, Dublin, Ireland; 11 Department of Microbiology, St. Vincent's, Sydney, New South Wales, Australia; 12 Division of Infectious Diseases, American University of Beirut, Beirut, Lebanon; 13 Infectious Disease Clinical Research Program, Uniformed Services University, Bethesda, Maryland, United States of America; 14 Department of Microbiology, Middlemore Hospital, Auckland, New Zealand; 15 Department of Infectious Diseases, Wellington Hospital, Wellington, New Zealand; 16 Department of Infectious Diseases, North Shore Hospital, Auckland, New Zealand; 17 Institute of Experimental Cardiology, Russian Medical State University, Moscow, Russia; 18 Departent of Medicine, VA Medical Centre, Durham, North Carolina, United States of America; 19 Infectious Disease Division, Medical University of South Carolina, Charleston, South Carolina, United States of America; 20 Infectious Diseases, Duke University Medical Center, Durham, North Carolina, United States of America; 21 Duke University Medical Center, Durham, North Carolina, United States of America; Virginia Commonwealth University, United States of America

## Abstract

The HACEK organisms (*Haemophilus* species, *Aggregatibacter* species, *Cardiobacterium hominis*, *Eikenella corrodens*, and *Kingella* species) are rare causes of infective endocarditis (IE). The objective of this study is to describe the clinical characteristics and outcomes of patients with HACEK endocarditis (HE) in a large multi-national cohort. Patients hospitalized with definite or possible infective endocarditis by the International Collaboration on Endocarditis Prospective Cohort Study in 64 hospitals from 28 countries were included and characteristics of HE patients compared with IE due to other pathogens. Of 5591 patients enrolled, 77 (1.4%) had HE. HE was associated with a younger age (47 vs. 61 years; p<0.001), a higher prevalence of immunologic/vascular manifestations (32% vs. 20%; p<0.008) and stroke (25% vs. 17% p = 0.05) but a lower prevalence of congestive heart failure (15% vs. 30%; p = 0.004), death in-hospital (4% vs. 18%; p = 0.001) or after 1 year follow-up (6% vs. 20%; p = 0.01) than IE due to other pathogens (n = 5514). On multivariable analysis, stroke was associated with mitral valve vegetations (OR 3.60; CI 1.34–9.65; p<0.01) and younger age (OR 0.62; CI 0.49–0.90; p<0.01). The overall outcome of HE was excellent with the in-hospital mortality (4%) significantly better than for non-HE (18%; p<0.001). Prosthetic valve endocarditis was more common in HE (35%) than non-HE (24%). The outcome of prosthetic valve and native valve HE was excellent whether treated medically or with surgery. Current treatment is very successful for the management of both native valve prosthetic valve HE but further studies are needed to determine why HE has a predilection for younger people and to cause stroke. The small number of patients and observational design limit inferences on treatment strategies. Self selection of study sites limits epidemiological inferences.

## Introduction

The HACEK group of bacteria (*Haemophilus* species, *Aggregatibacter* species, *Cardiobacterium hominis*, *Eikenella corrodens*, and *Kingella* species) are a small, heterogeneous group of fastidious, gram-negative bacteria that frequently colonize the oropharynx and have long been recognised as a cause of infective endocarditis (IE). These organisms have been historically reported as causing infection in <5% of patients of IE [1], [2], and 0.8–6% of patients in recent population-based studies [3]–[5].

Due to the relative rarity of HACEK endocarditis (HE), the clinical description and outcome has, of necessity, been derived from compilation of data from small case series and case reports [6]–[10]. These reports are limited by non-standardized data collection and selective reporting of patients. Consequently, the features of HE identified cannot be compared rigorously with other forms of IE.

The International Collaboration on Endocarditis Prospective Cohort Study (ICE-PCS) was designed to provide a large multi-national resource of prospectively collected, well defined patients of IE using a standardised data set. The objective is to improve understanding of the clinical characteristics and outcome of IE in a multi-national cohort of patients. In this report we describe the characteristics of patients with HE, and compare the risk factors, clinical characteristics, and outcomes of HE with IE caused by other pathogens (non-HE).

## Patients and Methods

### Database

ICE-PCS has been described previously [11], [12]. Briefly, participating members from 64 sites in 28 countries reported patients prospectively on a standard case report form from June 2000 through to September 2006. The case report form included 275 variables and was developed by ICE collaborators according to standard definitions [11]. All patients were included from sites that met performance criteria for participation. These criteria include the following: minimum enrolment of 12 patients per year in a centre with access to cardiac surgery; the presence of patient identification measures to ensure consecutive enrolment and to minimise ascertainment bias [11]; high quality data with query resolution.

### Ethical Statement

Initial institutional review board approval for ICE-PCS came from the Duke International Centre. All participating sites had institutional review board or ethical committee approval or a waiver and informed consent (verbal or written) or a waiver of consent from all patients based on local standards as required by the Duke Coordinating Centre.

### Study Sample

Patients in the ICE-PCS database were included in this study if they had definite or possible IE according to the modified Duke's criteria. HACEK isolates were identified and antibiotic susceptibility testing performed in the participating centres. Antibiotic therapy was decided by the treating physician at the individual study site.

### Definitions

Infective endocarditis was defined according to the modified Duke Criteria [13]. Infective endocarditis was considered to be left sided if no right sided (tricuspid or pulmonary valve) vegetations were present on echocardiographic examination, surgery, or autopsy. Community-acquired IE was defined as signs or symptoms of IE developing before hospitalization in a patient without extensive out-of-hospital contact with health care interventions or systems. Hospital acquired IE was defined as symptom onset and diagnosis occurring in a patient hospitalized for more than 48 hours.Health care–associated infection was defined as cases in which signs or symptoms consistent with infective endocarditis developed before hospitalization in patients with extensive out-of-hospital contact with health care interventions. Extensive out of hospital intervention included one or more of the following,(1) receipt of intravenous therapy, wound care, or specialized nursing care at home within the 30 days prior to the onset of IE; (2) visiting a hospital or hemodialysis clinic or receiving intravenous chemotherapy within the 30 days before the onset of IE; (3) hospitalization in an acute care hospital for 2 or more days in the 90 days before the onset of IE; or (4) residing in a nursing home or long-term care facility[14], [15].

Cancer was defined as any malignant neoplasm except basal or squamous cell carcinomas of the skin. The category “other chronic diseases” included connective tissue or rheumatologic disease, chronic liver or kidney disease, chronic neurological conditions, and other chronic infectious and inflammatory conditions. A diagnosis of heart failure was accepted on the basis of clinical evaluation performed by the care team and defined according to the New York Heart Association classification system [16]. Stroke was defined as an acute neurological deficit of vascular aetiology lasting more than 24 hours [17]. Systemic embolisation included embolisation to any organ including the skin. Valve surgery included all surgery performed on heart valves at any time during hospitalisation regardless of urgency. Rates of surgery and mortality include events that occurred during the index hospitalisation and one year follow-up. Repeat IE was defined as a further episode of IE fulfilling the modified Duke criteria. Confirmed relapse was defined as a repeat episode caused by the same microorganism on molecular analysis, as the preceding episode; confirmed new infection as a repeat episode caused by a different species or the same species but a different strain by molecular analysis; and possible relapse as repeat episode caused by a microorganism of the same species within 6 months of the initial episode without molecular analysis [18].

### Geographic regions

Geographic regions participating in ICE included the following: United States (10 sites), South America (9 sites from Argentina, Brazil, and Chile), Europe (27 sites from Austria, Croatia, France, Germany, Greece, Ireland, Italy, the Netherlands, Romania, Russia, Slovenia, Spain, Sweden, and the United Kingdom), Australia and New Zealand (9 sites), Asia and Middle East (8 sites in India, Israel, Lebanon, Malaysia, Saudi Arabia, Singapore, and Thailand,) and South Africa (1 site).

### Microbiological methods

Blood cultures and sensitivity testing was performed by accredited laboratories using standard methods. Sensitivity testing was most commonly those of the Clinical and Laboratory Standards Institute (CLSI)

### Statistical analysis

Continuous variables were represented as medians with 25^th^ and 75^th^ percentiles. Categorical variables were represented as frequencies and percentages of the specified group. Simple comparisons were made with the Wilcoxon rank-sum test or the chi square test as appropriate. For all tests, a *p* value of 0.05 or less was considered statistically significant. Missing data for each variable were excluded from the denominator. Variables found to have a simple association with the outcome of interest (*p*<0.10) were considered for the final multiple variable model in a stepwise fashion. The variables included in the final multiple variable adjusted regression model were selected based on a combination of statistical significance (*p*<0.05) and clinical judgment. The generalized estimating equation method was used to produce consistent parameter estimates that measure association between the incidence of outcome and clinical covariates while accounting for the correlation in treatment and outcomes of patients from the same hospital. Final parameter estimates were converted to ORs with corresponding 95% Wald CIs. All statistical analyses were performed using SAS version 9.2 (SAS Institute, Cary, NC, USA).

## Results

Seventy seven (1.4%) of 5591 patients diagnosed with IE in ICE-PCS had HACEK endocarditis (66 definite and 11 possible) and PVE was present in 27 (35%). The prevalence of HE differed significantly between the study sites (p = 0.009), with a low prevalence in North America and a high prevalence in Australia/New Zealand. The HE cases by region were: North America (5/992, 0.5%), South America (8/518, 1.5%), Australia/New Zealand (23/979, 2.3%), Europe (35/2806, 1.2%), Asia/Middle East (5/277, 1.8%), and Africa (1/19).

### Features of HE by species

The HACEK isolates were speciated in 76 (99%) cases with *Haemophilus* species the most common (40%) (Table 1). Of all HACEK species, only *Kingella* spp. was not associated with an episode of prosthetic valve endocarditis (PVE). PVE was more common in *A. actinomycetemcomitans* than *H. parainfluenzae* IE (10, 67% v 5, 18%; respectively p<0.01). Clinical manifestations of IE of more than 1 months duration were recorded more often in *A. actinomycetemcomitans* (8, 53%) and *Cardiobacterium* IE (6, 55%) than *H. parainfluenzae* (3, 11%; both p<0.01). Aortic valve vegetations were identified on echocardiography more commonly in *Cardiobacterium* IE (8, 89%) than in *H. parainfluenzae* (6, 32%; p<0.05) and *A. actinomycetemcomitans* (2, 29%; p<0.05) IE. Mitral valve endocarditis was common in *H. parainfluenzae* (10, 53%) and *A. actinomycetemcomitans* (6, 86%). Of the five cases with Oslers' nodes four occurred with *A. actinomycetemcomitans* IE.

**Table 1:** HACEK organisms isolated from definite and probable cases of HACEK endocarditis.

HACEK organisms	Number (%)
***Haemophilus*** ** spp.**	**31 (40)**
*Haemophilus parainfluenzae*	28 (36)
*Haemophilus* sp. other^a^	3 (4)
***Aggregatibacter*** ** spp.**	**26 (34)**
*Aggregatibacter actinomycetemcomitans*	15 (20)
*Aggregatibacter aphrophilus*	5 (6)
*Aggregatibacter paraphrophilus*	5 (6)
*Aggregatibacter segnis*	1 (1)
***Cardiobacterium*** ** spp.**	**11 (14)**
*Cardiobacterium hominis*	10 (13)
*Cardiobacterium valvarum*	1 (1)
***Eikinella corrodens***	**4 (5)**
***Kingella*** ** spp.**	**4 (5)**
*Kingella kingii.*	2 (3)
*Kingella denitrificans*	1 (1)
*Kingella* sp.	1 (1)
**HACEK** (not otherwise specified)	**1** (1)
**Total**	**77**

a not specified.

### Clinical features of HACEK and non-HACEK endocarditis

Baseline characteristics and predisposing factors of HE are shown in Table 2. The median age of patients with HE (47.4 years; IQR 35.6–57.1) was significantly lower than non-HE (60.5 years; IQR 45.3–72.7) and males predominated (56, 73%). Factors more commonly associated with HE than Non-HE endocarditis were Osler's nodes (7% vs 3%, p = 0.02) and vascular immunological phenomena (32% vs 20%, p = 0.008) and the presence of mechanical valves (30% vs 18%, p = 0.02). Factors less commonly associated with HE than non-HE endocarditis were health care provision (1% vs 24%, p<0.001), and diabetes mellitus (8% vs 18%, p = 0.02). There was no difference in the proportion with fever or splenomegaly between HE and non-HE, nor with native valve predisposition for IE or congenital heart disease (Table 2).

**Table 2:** Important features of HACEK endocarditis compared with all other causes of infective endocarditis in database*.

	HACEK Endocarditis n = 77	Non-HACEK Endocarditis n = 5514	*P* value
**Clinical Features**			
Median age (interquartile range), *y*	47.4 (35.6–57.1)	60.5 (45.3–72.7)	<0.001
Prosthetic valve endocarditis	27/77 (35)	1298/5514 (24)	0.07
Manifestations >1 month	18/77(23)	1174/5294(22)	0.68
Osler's nodes	5/71 (7)	132/5260 (3)	0.02
Conjunctival haemorrhages	6/72 (8)	214/5261 (4)	0.07
Vascular/immunologic evidence of endocarditis	25/77 (32)	1118/5514 (20)	0.008
**Risk factors**			
Diabetes mellitus	6/77 (8)	962/5417 (18)	0.02
Health care-associated	1/77 (1)	1349/5514 (24)	<0.001
Congenital heart disease	11/66 (17)	533/4813 (11)	0.16
Native valve predisposition	25/76 (33)	1609/5403 (30)	0.55
Mechanical aortic valve	12/77 (16)	504/5507 (9)	0.07
Bioprosthetic aortic valve	6/77 (8)	426/5507 (8)	0.9
Mechanical mitral valve	11/77 (14)	404/5504 (7)	0.03
Other	5/77 (7)†	604/5504 (11)	0.27
**Diagnosis**			
Blood culture growth	73/77 (95)	4586/5430 (84)	0.01
Other specimens culture positive	29/76 (38)‡	2787/5489 (51)	0.03
**ECHO findings**			
Intracardiac vegetations	53/75 (71)	4455/5383 (83)	0.01
Aortic Valve	26/53 (49)	1959/4455 (44)	0.49
Mitral Valve	25/53 (47)	2043/4455 (46)	0.9
Tricuspid Valve	1/52 (2)	578/4394 (13)	0.02
Other	4/53 (8)§	611/4455 (14)	0.23
New regurgitation	46/75 (61)	3124/5368 (58)	0.62
Paravalvular complications	15/75 (20)	1117/5354 (21)	0.85
**Outcome**			
Stroke	19/76 (25)	898/5410 (17)	0.05
Embolic stroke	10/18 (56)	648/780 (83)	0.008
Haemorrhagic stroke/intracranial haemorrhage	8/18 (44)	132/780 (17)	0.006
Congestive heart failure	11/74 (15)	1646/5397 (30)	0.004
Embolization, excluding central nervous system	15/73 (21)	1205/5399 (22)	0.79
Intracardiac abscess	14/75 (19)	721/5402 (13)	0.17
Mycotic aneurysm	3/74(4)	104/5351 (19)	0.15
Surgery	31/77 (40)	2433/5482 (44)	0.49
Median days in hospital (interquartile range)	23 (15–42)	28 (15–44)	0.19
In-hospital death	3/77 (4)	998/5508 (18)	0.001
Death within 1 year of admission	6/57 (11)**	1627/4208 (39)**	0.001

* Values are reported as *n/n (%)*, unless otherwise noted.

† Aortic valve- homograft 1, unknown repair 1; mitral valve – repair with prosthesis 1, other 2. TEE = Transesophageal ECHO.

‡ Specimens that were culture-positive were heart valve (20), joint fluid (2), pacemaker wire (1), urine (1) and other (5) §Myocardial wall 2, chordae 1, intracardiac device 1.

** 1 year mortality data was available on 57(74%) of HE and 4208(76%) of non-HE subjects.

### Transfers from another facility

There was no difference in the number of cases transferred from another facility between HE (30, 39%) and non-HE (2288, 41%; p = 0.6). In HE there were more cases transferred with native valve endocarditis (24, 80% vs 23, 49%; p = 0.01 ), new or worsening murmurs (20, 67% vs 19, 40% p = 0.008), regurgitation on echocardiography (23, 77% vs 23, 49%; p = 0.02), and need for valvular surgery (aortic valve 11, mitral valve 12) ( 23, 77% vs 8, 17% p<0.001 ) compared with those directly admitted. There was a borderline significant increase in stroke among transferees (11, 38% vs 8, 17%; p = 0.06) and CHF (7, 32% vs 4 9%; p = 0.10) and no difference in the numbers of cases with symptoms longer than 1 month (5, 17% vs 13, 28% p = 0.41) or length of hospital stay (median 23 IQR 15–42 vs median 27 IQR 14–42, p = 0.56).

### Diagnosis

Blood cultures were drawn in all 77 patients with HE. Three of four patients with negative blood cultures had received antibiotics in the previous seven days. Additional culture positive sites were heart valves (20), joint fluid (2), pacemaker wires (1), urine (1), and other (5). One patient was diagnosed by PCR of infected tissue. Echocardiography was performed in 97% of HE (transthoracic only 15, transesophageal only 9, both transthoracic and transesophageal 51). Vegetations were identified in a lower proportion in HE than non-HE (71% vs 83%, p = 0.01) (Table 2). There was no difference in the proportions of mitral and aortic valve vegetations identified between HE and non-HE. Only 1 case of tricuspid valve endocarditis was recorded in HE. New regurgitation and paravalvular complications were not significantly different from non-HE (Table 2).

### Antimicrobial susceptibility testing

The causative organisms are shown in table 1. Of the isolates tested 24/25 (96%) were penicillin susceptible (1 resistant strain of *A. aphrophilus*), 48/49 (98%) were ampicillin susceptible (1 resistant strain, of *A. aphrophilus*), 50/50 (100%) were ceftriaxone susceptible, and 30/32 (94%) were gentamicin susceptible (2 resistant strains of *H. parainfluenzae*).

### Treatment

Antimicrobial therapy was reported in 50 (65%) patients. Of these 37 (74%) were treated with ceftriaxone (in combination with an aminoglycoside in 17 and ampicillin in 6) , 6 with a penicillin derivative (ampicillin in 3, penicillin G in 2, and penicillinase-resistant penicillin in one,in combination with an aminoglycoside), and 3 with cefazolin/cefalothin (in combination with an aminoglycoside), and 4 unspecified. All cases of HE were treated with antimicrobial agents that would be active as predicted by susceptibility testing. Cardiac surgery was performed on 31 (40%) patients a median of four days (IQR 1–19) after admission. The aortic valve was replaced in 17 patients, mitral valve in 13, tricuspid valve in one and an intracardiac device was removed in one patient.

### Outcomes

The in-hospital mortality of HE was less than one quarter that of the non-HE (3, 4% vs 998,18%; p<0.001). Of the three HE deaths with one had been treated surgically. Heart failure was significantly less frequent in HE than non-HE (15% vs. 30%, p = 0.004). Stroke complicated a higher proportion of cases with HE than non-HE (25% vs. 17%, p = 0.05) and there was a relative excess of haemorrhagic stroke over embolic stroke in HE (44% vs 17%, p = 0.006). The presence of a stroke increased the length of stay by 20 days despite occurring in a significantly younger age group (Table 3). On multivariable regression analysis the independent factors associated with stroke were increasing age in 10 year intervals (OR 0.62; CI 0.49–0.90; p<0.01) and mitral valve vegetations (OR 3.60; CI 1.34–9.65; p<0.01). Eleven of 25 (44%) cases of HE with mitral valve vegetations suffered a stroke compared with 484/2009 (24%) in non-HE (p = 0.03). The frequency of systemic embolization, excluding central nervous system, intracardiac abscess and mycotic aneurysm were not significantly different in HE than non-HE (Table 2).

**Table 3:** Univariate and multivariate analysis for the risk of stroke in HACEK endocarditis*.

	No stroke	Stroke	Univariate analysis	Multivariate analysis
	n = 57†	n = 19†	*P* value	Odds Ratio (95% confidence interval)
Prosthetic valve endocarditis	20/57 (35)	7/19 (37)	0.9	
Median age (interquartile range), *y*	51 (38–60)	41 (25–54)	0.02	0.62; (CI 0.49–0.90) *P*<0.01
>1 month from 1st manifestation	13/57 (23)	5/19 (26)	0.8	
Conjunctival haemorrhages	1/52 (2)	5/19 (26)	0.001	
Osler's nodes	4/52 (8)	1/18 (6)	0.8	
Vascular/immunologic evidence of endocarditis	13/57 (23)	11/19 (58)	0.004	
Aortic valve vegetation	22/37 (59)	4/16 (25)	0.02	
Mitral valve vegetation	14/37 (38)	11/16 (69)	0.04	3.60 (CI 1.34–9.65) *P*<0.01
Aortic valve surgery	14/21 (67)	4/9 (44)	0.26	
Mitral valve surgery	6/21 (29)	7/9 (78)	0.01	
Embolization	10/56 (18)	5/17 (29)	0.3	
Mycotic Aneurysm	0/57 (0)	3/19 (16)	0.003	
Median days in hospital (interquartile range)	22 (14–34)	42 (21–60)	0.002	
In-hospital death	2/57 (4)	1/19 (5)	0.7	

* Values are reported as *n/n (%)*, unless otherwise noted.

† There was missing data for stroke on one patient.

At one year follow-up, three additional cases of HE had died (heart failure 1, unrelated causes 1, unknown 1); however the cumulative death rate was significantly lower than non-HE (6,11% vs 1627, 39%; p<0.001) (Table 2). Four cases had undergone valvular surgery; three had been treated medically and one surgically.

There was one possible relapse 4 months after completing therapy with an unspecified HACEK organism. This organism was not available for further speciation. In addition one patient with HE had another episode of endocarditis with a methicillin susceptible S. aureus.

#### HACEK native and prosthetic valve endocarditis

Comparison of the clinical features of native valve and prosthetic valve HE demonstrated that native valve HE occurred at an older age (median 56.3 (range 41–67) vs median 43.8 (range 32–54) years, p = 0.003), and that a higher proportion had Osler's nodes (5, 20% vs 0, 0%; p = 0.002) and systemic embolization (10, 37% vs 5, 12% v; p = 0.01) than prosthetic valve HE (Table 4). There was no significant difference in the proportion with stroke (7, 26% vs 11, 24%; p = 0.85), or congestive heart failure (5, 19% vs 6, 14%; p = 0.74) or surgical treatment (8, 30% vs 22, 45%; p = 0.23) or length of median hospital stay between these groups.

**Table 4:** Comparison between the features of native valve HACEK endocarditis and prosthetic valve HACEK endocarditis*.

	Native valve Endocarditis	Prosthetic valve endocarditis	*P* value
	n = 47	n = 27	
Duke diagnosis definite	43/47 (92)	21/27 (78)	0.01
Median age (interquartile range), *y*	43.8 (32–54)	56.3 (41–67)	0.003
Osler's nodes	0/43 (0)	5/25 (20)	0.002
Worsening of old murmur or presence of new murmur	30/47 (64)	8/27 (30)	0.005
ECHO evidence of new regurgitation	36/47 (77)	10/27 (37)	<0.001
Intracardiac vegetations	36/46 (78)	15/27 (56)	0.02
*Aortic valve*	17/36 (47)	9/15 (60)	0.4
*Mitral valve*	18/36 (50)	7/15 (47)	0.8
Aortic valve surgery	11/22 (50)	7/8 (88)	0.06
Stroke	11/46 (24)	7/27 (26)	0.85
*Embolic*	6/11 (55)	4/6 (67)	1.0
*Intracerebral haemorrhage*	5/11 (45)	2/6 (33)	1.0
Other systemic Embolization	5/43 (12)	10/27 (37)	0.01
Congestive heart failure	6/44 (14)	5/27 (19)	0.13
Surgery	21/47 (45)	8/27 (30)	0.23
In-hospital death	2/47 (4)	0/27 (0)	0.28
Median days in hospital (interquartile range)	21 (12–37)	24 (17–43)	0.2
Death within 1 year of admission	3/33 (9)†	1/22 (5)†	0.8

* Values are reported as *n/n (%)*, unless otherwise noted.

† 1 year mortality data was available on 33 (70%) subjects with native valve HE and 22(81%) subjects with prosthetic valve HE.

Of those with PVE 8 (30%) required surgical treatment and 19 (70%) were treated with medical therapy alone. There were no in-hospital deaths in either treatment group. Of the 24 PVE patients with 1 year follow-up data, there was one death (cause unknown) and three who required cardiac surgery in the medically treated group, but no deaths, relapses or further surgery requirement in the surgically treated group. By comparison, among those with native valve HE, there were two in-hospital deaths and 1 death with-in the 1 year follow-up period.

## Discussion

This report describes the findings of a large series of HE and non-HE cases of bacterial endocarditis reported in a standardised manner in which geographic distribution, frequency of clinical features, risk factors and outcomes have been compared. Both groups were subject to referral bias from transfers to the study centres [19], [20], but this is unlikely to confound these comparisons as the proportion transferred was very similar in the two groups, and the pattern of features of the transferees with HE was similar to that reported in the ICE cohort. However the frequency of some clinical features are influenced by transfers between hospitals, and the results need to be interpreted in the light of this limitation.

The marked geographic difference in the prevalence of HE (10-fold) between the highest (New Zealand) and the lowest countries (United States of America) confirms the findings of an earlier, smaller sample of cases in the ICE cohort [11]. The range is similar to the range of 0.8–6.1% reported in recent single and multi-centre studies. [3]–[5]. The high prevalence of HE in New Zealand is unlikely to be due to referral patterns given the low proportion transferred from another facility. Other possible reasons for the variation include the prevalence of risk factors such as frequency of prosthetic devices [12], oral health [21], transmission pathways of HACEK organisms within populations [22], regional health care access, and diagnostic bias.

Some clinical features varied with the causative species. *H. parainfluenzae* was the commonest cause of HE, as has been reported in population based studies [23]. *H. parainfluenzae* endocarditis was less likely to have an insidious onset than both *A. actinomycetemcomitans* and *C. hominis* confirming previous reports [7]. *C. hominis* was strongly associated with aortic valve infection and *A. actinomycetemcomitans* endocarditis was a frequent cause of PVE, and vascular immunological manifestations [6], [7], [9], [10]. Despite this, we found that HE has sufficient important clinical features in common that distinguish it from non-HE to retain clinical usefulness. These features include younger age of presentation, community acquisition, a higher proportion with vascular/immunological manifestations, a lower proportion with co-morbidities and an excellent outcome.

Prosthetic valve endocarditis was common (35%), although the prevalence of HACEK PVE was not significantly higher than non-HE PVE in this study (p = 0.07). However, the comparator includes a heterogeneous group of organisms with variable propensity to cause PVE. For example, previous studies of the ICE cohort have found the proportion of PVE in *Staphylococcus aureus* IE to be 16%, viridans streptococcal IE to be 15%, and coagulase-negative staphylococcal IE to be 32% [11]. Thus it appears that HACEK organisms have a predilection for prosthetic valves. This finding is more marked in late PVE (>1 year after surgery) as HE causes late PVE in a large majority of cases [12], but only about half of cases of *S. aureus* and coagulase-negative staphylococci PVE, suggesting mechanical valves are a particular risk for HE [17]. In addition this study may underestimate the true proportion with PVE because of the high number of native valve HE cases transferred from other centres. Pre-existing native valve and congenital cardiac abnormalities were common in HE but there was no significant increase in these conditions compared with non-HE. Previous studies have suggested these risk factors may occur more frequently in HE, but this will be subject to changes in the epidemiology of native valve lesions such as rheumatic fever and the widespread availability of cardiac surgery [22].

Most cases of HE were treated with a third generation cephalosporin and a minority with ampicillin with or without an aminoglycoside; however there were insufficient cases to correlate outcomes with these recommended regimens [24], [25]. The incidence of penicillin resistant strains was limited to an isolate of *A. aphrophilus* species. β-lactamase producing strains of *C. hominis* have been reported but not in *A. actinomycetemcomitans* to our knowledge [26]. The proportion of all HE cases requiring cardiac surgery (40%) was similar to non-HE and to that reported in the literature [12], [27]. However in PVE the requirement for surgery was lower (30%) which compares favourably with published rates for PVE overall (49%) [12], [27]. The favourable outcome of both medically and surgically treated HACEK PVE demonstrates that HE is readily controlled and treated with antimicrobial agents despite the presence of a prosthetic valve.

The outcome of HE was excellent overall with an in-hospital mortality of 3% which is less than one quarter of the mortality for non-HE and one sixth that of *S. aureus* endocarditis [28]. Heart failure was diagnosed in 15% of cases compared with 30% in non-HE, and 37% reported for *S. aureus* endocarditis [27], [28]. The younger age group and lack of co-morbidities, in addition to pathogen-specific characteristics may favour a good in-hospital and 1 year outcomes. These results would not be affected by survivor bias given the very high survival rate of HE. With respect to PVE, the numbers were too small to make meaningful comparison for other major complications including stroke, congestive heart failure and abscess formation.

The major complication of HE was stroke (25%), and this complication almost doubled the length of hospitalisation. This figure over-represents the true incidence of stroke in HE as there was an increased frequency of stroke in those transferred from other facilities. Nevertheless stroke is conspicuously common in HE compared with non-HE, and the reported frequency in *S. aureus* endocarditis (20%) and viridans streptococcal IE (8%) in the ICE cohort [29]. Mitral valve IE was an important risk factor for stroke as reported previously, but organism specific effects on the nature of the vegetations in HE may also make a significant contribution to the prevalence of stroke, as 44% of patients with mitral valve HE suffering a stroke compared with 24% of non-HE. This may be related to the long antecedent history with organisms such as *A. actinomycetemcomitans* [7].

While embolism was the predominant cause of stroke in HE there was relative excess of haemorrhagic stroke. The reasons for this are not clear but it is possible micro-vascular/immunological manifestations of IE which were significantly more frequent in HE than non-HE and might contribute to the development of cerebral microbleeds which are a strong predictor of subsequent intracranial haemorrhage [30]. Anticoagulant therapy is unlikely to contribute to the occurrence of stroke but may increase the conversion of embolic to haemorrhagic events. [31].

There are several additional limitations of this study. Because of small numbers both possible and definite cases were included to increase statistical power. Despite this there were a limited number of cases of HACEK endocarditis and the observational design and long-term follow-up limited to one year limits our ability to draw any firm conclusions regarding optimal antimicrobial therapy or surgical treatment strategies. Furthermore the self selection of centres to participate in the ICE study, and the heavy weighting toward Europe, North America and Australasia with few sites in Asia, and Africa has meant that the population sample may not be representative of any specific region. Thus important geographical differences may have been missed and any epidemiological inferences from this study are limited.

Our findings suggest that there is sufficient similarity in presentation and outcome to justify considering the HACEK organisms as a group at present. Despite the high prevalence of stroke, HE has a remarkably low mortality rate, suggesting that current antibiotic therapy with surgery when needed, is very effective. The reasons why HE shows apparent disparities in geographical distribution, occurs in a younger age group, has a propensity to infect prosthetic valves, and is associated with a high incidence of stroke are worthy of further investigation.
